# Household Wastes as Larval Habitats of Dengue Vectors: Comparison between Urban and Rural Areas of Kolkata, India

**DOI:** 10.1371/journal.pone.0138082

**Published:** 2015-10-08

**Authors:** Soumyajit Banerjee, Gautam Aditya, Goutam K. Saha

**Affiliations:** 1 Department of Zoology, 35, BC Road, University of Calcutta, Kolkata 700019, India; 2 Department of Zoology, The University of Burdwan, Burdwan 713104, India; National Taiwan Ocean University, TAIWAN

## Abstract

Porcelain and plastic materials constitute bulk of household wastes. Owing to resistibility and slow degradability that accounts for higher residence time, these materials qualify as potential hazardous wastes. Retention of water permits these wastes to form a congenial biotope for the breeding of different vector mosquitoes. Thus porcelain and plastic wastes pose a risk from public health viewpoint. This proposition was validated through the study on the porcelain and plastic household wastes as larval habitats of Dengue vectors (*Aedes* spp.) in rural and urban areas around Kolkata, India. The wastes were characterized in terms of larval productivity, seasonal variation and a comparison between urban and rural areas was made using data of two subsequent years. The number of wastes positive as larval habitats and their productivity of *Aedes* spp. varied among the types of household wastes with reference to months and location. Multivariate analysis revealed significant differences in the larval productivity of the household wastes based on the materials, season, and urban–rural context. Results of Discriminant Analysis indicated differences in abundance of *Ae*. *aegypti* and *Ae*. *albopictus* for the urban and rural areas. The porcelain and plastic wastes were more productive in urban areas compared to the rural areas, indicating a possible difference in the household waste generation. A link between household wastes with *Aedes* productivity is expected to increase the risk of dengue epidemics if waste generation is continued without appropriate measures to limit addition to the environment. Perhaps, alternative strategies and replacement of materials with low persistence time can reduce this problem of waste and mosquito production.

## Introduction

Plastic and porcelain wastes of household origin qualify as hazardous materials owing to their resistance to physical and chemical factors and slow degradability [1]. As a result, porcelain and plastic wastes may interfere with natural processes and influence environmental quality. In absence of suitable management, porcelain (including glass) and plastic wastes sustain pathogens and parasites of medical importance [2], posing concern from public health viewpoint [3,4] in different regions of the world [5–7]. The organic residues and entrapped water in porcelain (including glass) and plastic wastes create suitable biotope for breeding of vector mosquitoes, particularly *Aedes* spp. [8–10]. Theshape of the containers and residence time in the environment determine the quality of porcelain (glass) and plastic wastes as larval habitats of *Aedes aegypti* and *Aedes albopictus* [11,12]. This observation justifies porcelain and plastic wastes as contributors to breeding of dengue vectors and thus increases the corresponding risk of dengue transmission.

Dengue and Chikungunya are examples of mosquito borne viral diseases posing concern for public health worldwide, in the tropics and subtropics [13,14]. Monitoring of vectors of these diseases is necessary for predicting the population variations and intervention of the vector population. Thus assessment of the prospective breeding grounds of the vector mosquitoes forms an integral part of management of dengue and chikungunya [15,16]. Linking household wastes with the mosquito breeding enable characterization and classification of these wastes as key larval habitat of *Aedes* mosquitoes [12,17]. Household waste generation varies in urban and rural background, owing to characteristic population density, social, economic and environmental factors [18–21]. The distinction between the urban-rural areas in Kolkata, India is based on density of human settlements and source of livelihood. Consequently, the contributions of wastes as larval habitats are expected to vary according to rural and urban background [22–24]. In recent years, dengue and chikungunya outbreak in India is recorded mostly from urban areas [25–27], with few reports from rural areas [28,29]. With increased usage of plastic in various forms, the possible wastes generation in the rural areas cannot be ruled out [30–33]. Based on these observations and propositions, an attempt was made to evaluate the differences in the pupal productivity in rural and urban areas using Kolkata, India as the geographical study area. Earlier studies from eastern India including Kolkata, indicate expansion of the geographical range of dengue vectors [34,35]. While dengue epidemics have been largely restricted to urban areas, expansion of geographical range increases chance of dengue epidemics in rural areas. A comparative study of abundance of dengue vectors in rural and urban areas will enable highlighting the role of porcelain and plastic wastes as contributors to the sustenance of dengue vector populations. Apart from supplementing information to develop strategies for source reduction of the breeding habitats, the results of the study will enable predictions about possible expansion of the geographical boundaries of dengue and chikungunya vector mosquitoes in Kolkata, India and its adjoining areas.

## Material and Methods

### Study area

Following identification and subsequent screening of household hazardous waste containers as *Aedes* larval habitats, sampling studies were carried out from selected sites of Kolkata and adjoining rural areas of eastern India. Each sampling site within the study area included four sampling spots each from the urban and rural areas. The urban sites considered for the present study included Baranagar (22°38'36"N, 88°21'55" E), Ballygunge (22°32'0"N, 88° 22' 0" E), Chetla (22°31'0"N, 88°20'24"E), and Patuli (22°47’22”N, 88°38’78”E), Kolkata India; the rural spots consisted of Serampore (22°75’00”N, 88°34’00”E), Baidyabati (22°79’00” N, 88°32’00”E), Singur (22°81’00” N, 88°23’00”E) and Haripal (22°83’33” N, 88°11’67”E) of district Hooghly adjacent to Kolkata. In the present study, the areas where there is a population of minimum 5,000 individuals with a density of 400 persons per sq. km and 75% of the males were engaged in non-agricultural pursuits, were denoted as urban areas (viz. Baranagar, Ballygunge, Chetla, and Patuli) whereas areas having a population of maximum 15,000 individuals with a density of <400 individuals/Km^2^ and agriculture as the chief source of livelihood followed by fishing, cottage industries, pottery etc were categorized as rural (Serampore, Baidyabati, Singur and Haripal).

### Sampling procedure

No specific permissions were required for any locations / activities for the study. The GPS coordinates are being added in the Materials and methods section. The field studies did not involve endangered or protected species both in the urban and rural sites. In each month between July 2009 and December 2010, random stratified sampling was employed to monitor prospective *Aedes* larval habitats in the study area (Table 1). The study included survey of the urban (U) and rural (R) areas based on 4 sites (1–4) in each area. Four selected spots of equal space (~100m^2^) formed each site, under which randomly chosen 20 sub-spaces were surveyed for the presence of porcelain and plastic waste containers. In each spot within the study area, the *Aedes* mosquito larval habitats [11] were chosen randomly, based on two broad types, porcelain (including glass) and plastic waste containers. The sampling was carried out using WHO methods and following Krebs [36] and Focks & Alexander [11]. A total of 80 numbers of each habitat (cluster X sub- space) was considered per sampling spot per month. In the context of the sampling method, the term larval habitat denoted the different household non-reusable and non-degradable containers of either porcelain (including glass) or plastic material that was used by the *Aedes* for breeding. The different porcelain and plastic containers of household hazardous waste were checked both outdoor and indoor the human settlements for the *Aedes* immature. The features of the habitats surveyed are presented in Table 2. Each of the habitats was sampled either using an inverted glass pipette (100ml) fitted with rubber teats or emptying the whole contents in a glass beaker (500ml), according to the aptness of the habitats. There were cases where other species of vector mosquitoes (viz. *Culex* spp., *Anopheles* spp.) were encountered but for the present study, the number of individuals of *Ae*. *aegypti* and *Ae*. *albopictus* were considered only for analysis.

**Table 1:** Outline of design and objective of the study.

Attributes	Details	Remarks
Study area	Two	Urban and Rural
Sampling sites	Eight	
	Urban: U1, U2, U3, U4	U1- Baranagar, U2- Ballygunge, U3- Chetla, and U4- Patuli
	Rural: R1. R2, R3, R4	R1- Serampore, R2- Badidyabati, R3 -Singur and R4- Haripal
Habitats	Two categories	Plastic and porcelain containers
Study period	Two years	2009–2010
Sampling months	Six; July—December	Post monsoon months
Total habitats sampled	Eighty habitats of each type over the period of study in each area	80 x 2 x 8 x 2 x 2 x 6 = 30720
Observation	Larva and pupa, collectively considered as immature were sampled; reared under laboratory conditions for identifying species and sex	Waste containers having either *Ae*. *aegypti* or *Ae*. *albopictus* individually were only taken into account; cases where both the species occurred were excluded
Analysis	ANOVA; Discriminant function analysis	To comment on species specific variation habitat and area wise in number of positive habitats and abundance; Similarity pattern of house hold waste based on the abundance
Hypothesis tested	Variation of house hold hazardous wastes in urban and rural context and their linkage with mosquito productivity	Detection and prioritization of house hold generated hazardous wastes as key mosquito habitats

**Table 2:** Characteristics features of household hazardous materials (HHM), plastic (PL)and porcelain (glass) (GLP) wastes that were considered as potential *Aedes* mosquito larval habitats in the survey in Kolkata and rural areas of adjacent districts and were found positive for the *Aedes* mosquitoes. Figures in parenthesis reveal the percentage of the containers found positive for the mosquitoes. The containers were surveyed with respect to human dwelling and categorized as: located outside human house = O, located inside human houses = I.

House hold hazardous material (HHM)	Location (L)	Common name and abbreviation used in the text	Utility	Diameter/ Length (in cm)	Height (in cm)	Breadth (in cm)	Water holding capacity (ml)	Positive containers *Ae*. *aegypti*	Positive containers *Ae*. *albopictus*
PL	O	Cup (OP1)	Drinking	4–4.5	3.8–4.5	NA	<100	1184 (15.42)	1155 (15.04)
PL	O	Broken Bucket (OP2)	Washing	18–30	15–30	NA	>250	248 (3.23)	417 (5.43)
PL	O	Bowl(OP3)	Drinking/utensil	8.0–11.0	3–6.5	NA	100–250	686 (8.93)	700 (9.11)
PL	O	Short container (OP4)	Cosmetic/baby food/utensil	4.5–26	5.0–12.0	NA	<100	309 (4.02)	307 (4.0)
PL	O	Box (OP5)	Utensil/carrying eatables	5.0–29.0	4.2–8.4	4.1–20.0	100–250	526 (6.85)	295 (3.84)
PL	I	Cup (IP1)	Drinking	3.8–4.5	3.8–4.5	NA	<100	925 (12.04)	1131 (14.73)
PL	I	Bowl (IP2)	Drinking/utensil	7.2–10.0	3–6.5	NA	100–250	452 (5.89)	303 (3.95)
PL	I	Short container (IP3)	Cosmetic/baby food/utensil	2.0–8.0	5.0–12.0	NA	<100	518 (6.74)	539 (7.02)
GLP	O	Sink (OPR1)	Toiletries	26–55	10.0–15.0	20–38	>250	276 (57.5)	282 (58.75)
GLP	O	Vase (OPR2)	Decoration			NA	100–250	1089 (68.06)	1495 (93.44)
GLP	O	Soup Bowl (OPR3)	Utensil	6.1–18.5	5.0–7.0	NA	<100	1103 (45.96)	1588 (66.17)
GLP	O	Broken showpiece (OPR4)	Decoration				<100	1040 (43.33)	859 (35.79)
GLP	I	Sink (IPR1)	Toiletries	24–55	10.0–17.0	22–40	>250	335 (41.88)	529 (66.13)

### Observations

From each of the positive larval habitats, the immature comprising of the larva and pupa were sampled, put in sample containers (100ml sample container Tarsons®) and brought to the laboratory to note the number of immature collected. Following counting, the immature were allowed to develop to adults in the sample containers marked with the site and area of collection. Depending on the density of the immature (<100 individuals), the sample containers were supplemented with 5–15 grains of fish food (®Tokyu fish food) in adequate amount of water ~ 30 mL –50 mL. The water was changed regularly and the adult emerging was counted further. For densities greater than 100 individuals collected from a larval habitat, immature were reared in plastic trays (15 X 11 X 3 inches) to adult stage for identification to the species level. The sex and species of the adults were identified based on appropriate keys [37,38]. In case of larval habitats that were positive for both the species, data was recorded against a single species (based on relative abundance) to avoid pseudoreplication [39]. Thus larval habitats were categorized for only two species separately. The discarded containers harbouring either *Ae*. *aegypti* or *Ae*. *albopictus* individually were taken into account only. There were cases where both the species occurred in tandem in a container, but those samples were included as positive for a particular species based on relative density to exclude the possibility of pseudo replication [39]. The problem of pseudo replication arises due to lack of appropriate replicate (randomization and interspersion) or the replicates fail to be statistically independent. For the present study the smallest experimental unit to which a treatment is independently applied is a single porcelain (glass) or plastic waste. Thus a single porcelain (including glass) or plastic waste could be considered only once as a replicate, either for *Ae*. *albopictus* or for *Ae*. *aegypti*. Considering the single unit for larval habitat of both would increase the number of sampling units and add error to the analysis. The co-occurrence of *Ae*. *aegypti* and *Ae*. *albopictus* in the habitats, is a mutually inclusive phenomenon where one species may occur at the same time and in the same habitat with the other. The density of *Ae*. *aegypti*was higher in the plastic containers. Hence in cases where the both species coexist in the same container (viz. plastic) depending on the relative density, the species with higher density were included to a particular category. However, it was noted that in porcelain (glass) containers, in cases of ‘both’, relative density of *Ae*. *aegypti* and *Ae*. *albopictus* were more or less equal. In such cases, numbers of positive habitats were assigned equally between them.

### Statistical analysis

To comment on the habitat and area, data on positive larval habitats and abundance were subjected to three-way factorial ANOVA [40] using habitats, area and month as variables. Further to reveal species specific variation, the data on immature abundance were analyzed for five-way factorial ANOVA using species, month, habitat, area and location as variables. The data on the positive larval habitats, relative abundance of larvae and pupae in house hold generated wastes of porcelain and plastic containers were subjected to discriminant function analysis [41] to comment on the differences in immature abundance in urban- rural context and months. Discriminant function analysis (DA) is a multivariate procedure that enables segregation among target variables using certain explanatory variables. DA is reverse of multivariate analysis of variance (MANOVA) in the sense that dependent variables are the groups (*Aedes* spp. productivity) and the predictor or input variables (urban-rural gradient, months) are the independent variables. In MANOVA, the independent variables are the groups and the dependent variables are the predictors. The DA is divided into two phase study, beginning with, first, the test of significance for a determined number of Discriminant functions, followed by, second—the categorization. In Discriminant Analysis (DA) [41] classification of the heterogeneity in the data based on particular parameters can be carried out so as to segregate the variables based on observed data. This helps to determine if there is any significant difference among the different groups with regards to the various parameters considered. In the present study, the urban-rural gradient and months were considered as predictor variables to discriminate the productivity of *Ae*. *aegypti* and *Ae*. *albopictus*.

The results of DA would enable portraying the productive months and sites in terms of abundance of immature *Aedes* mosquitoes thereby highlighting the differences among the response variables. The statistical analyses were performed using SPSS ver. 10 software and *XL*STAT [42].

## Results

**Fig 1:**
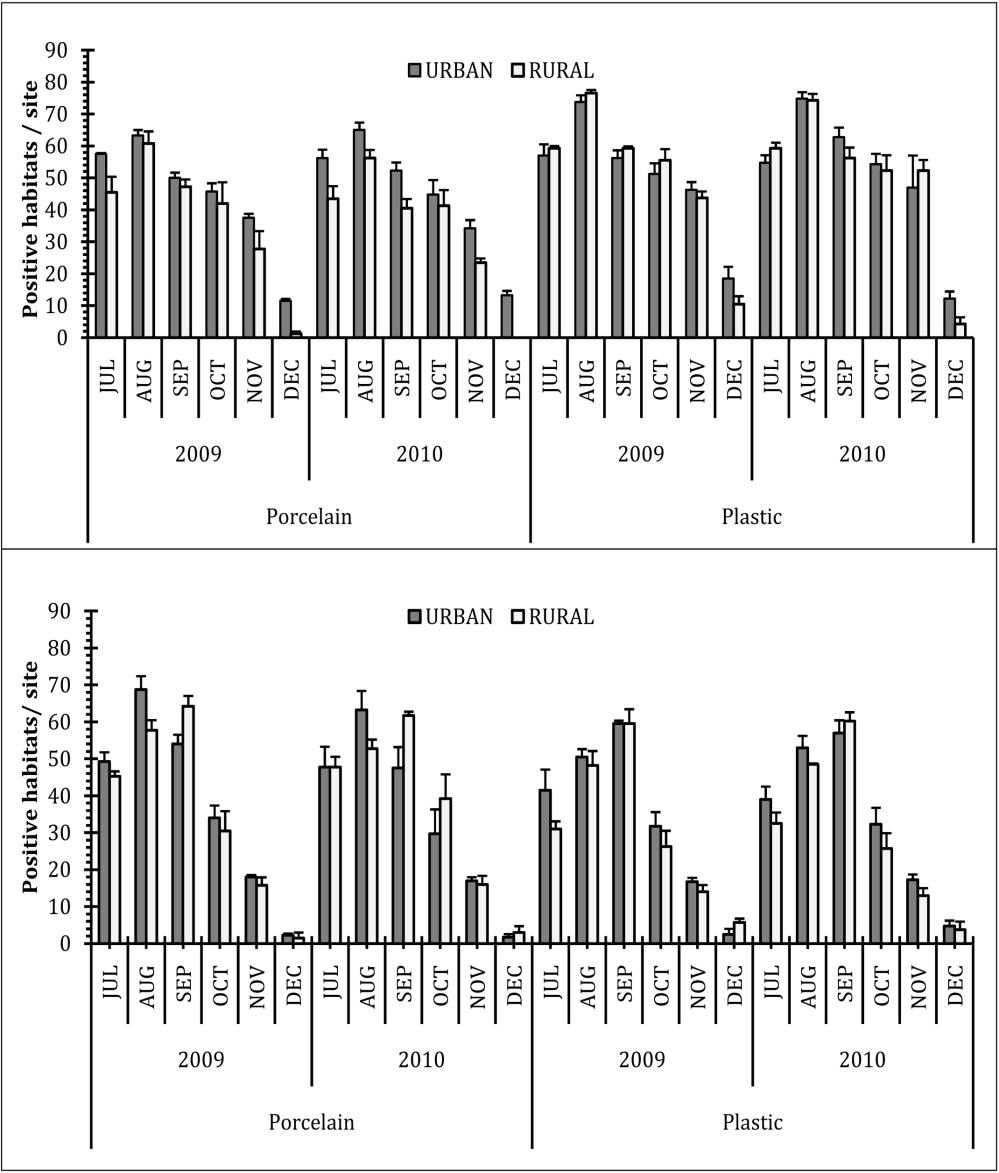
Different household hazardous disposable plastic and porcelain containers observed to be positive (Mean± SE) for either (a) *Ae*. *aegypti* or(b) *Ae*. *albopictus* during the two years course of study (July-December of 2009 and2010)in Kolkata and adjoining areas of West Bengal, India. (Sample size n = 20 numbers of each of the type of habitats. Cumulative total positive is presented under the two main categories—porcelain and plastic. Four each of urban and rural sites were considered for sampling in each month.

**Fig 2:**
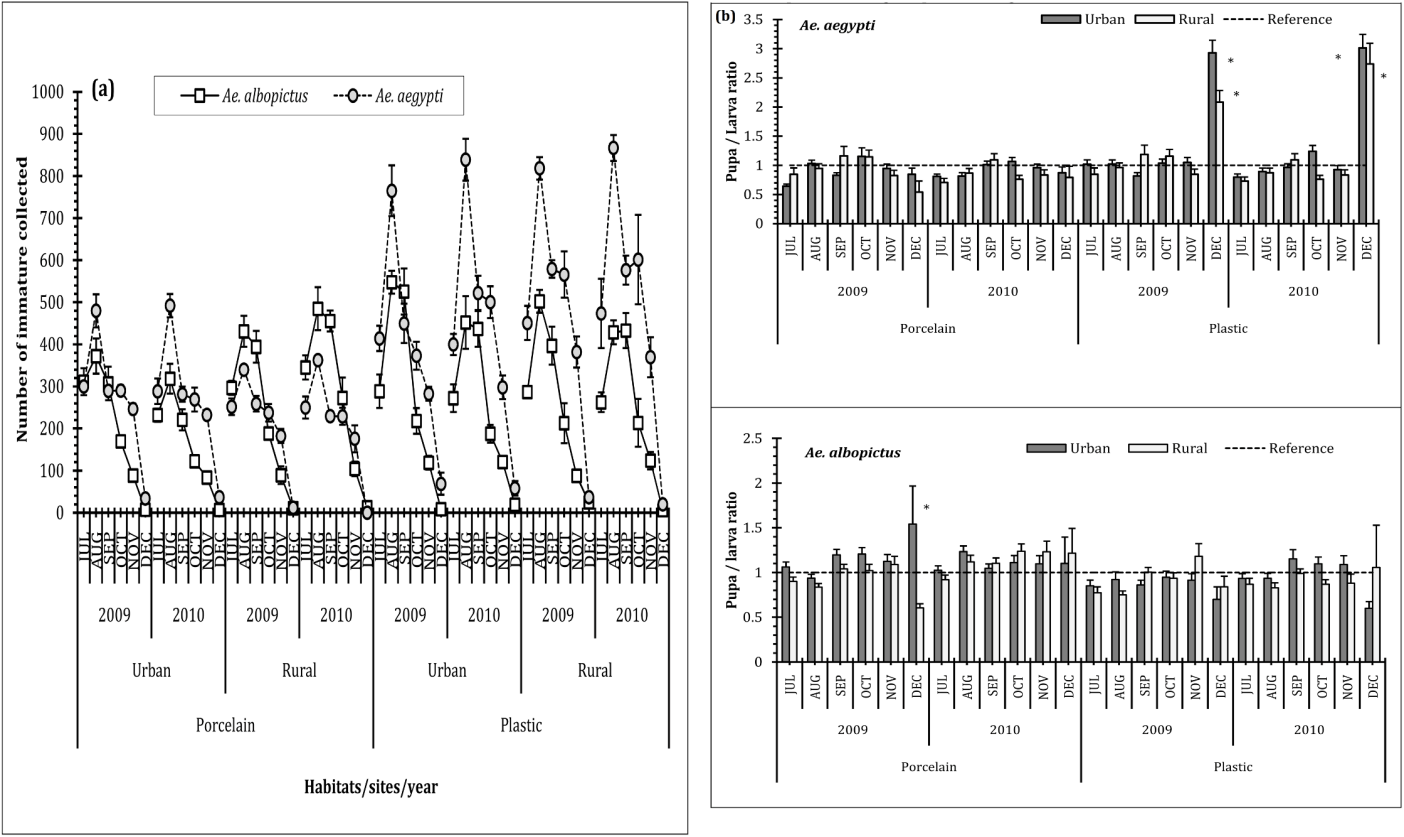
The abundance (Mean ± SE) of *Ae*. *aegypti* and *Ae*. *albopictus* immature (a) and the pupa/ larvae ratio (Mean ± SE) (b) in various plastic and porcelain household disposed containers, during the two years course of study (July-December of 2009 and 2010) in Kolkata and adjoining areas of West Bengal, India. (Sample size n = 20 numbers of each of the type of habitats. Cumulative total positive is presented under the two main categories—porcelain and plastic. Four each of urban and rural sites were considered for sampling in each month. The total immature (larva and pupa) from the positive habitats of different types of porcelain and plastic containers are shown here. In figure b, the pupa/ larva ratio per habitat with reference line representing equality of the two morphs. The * sign represents significant deviations from 1.

The number of habitats recorded positive for the species *Ae*. *aegypti* and *Ae*. *albopictus*, was found to vary with the type of habitat and material of the house-hold generated wastes (Table 2). In terms of positive number of breeding sites for the immature, porcelain (glass) objects (OPR1, OPR2, OPR3, OPR4, and IPR1) were more productive for *Ae*. *albopictus*. The plastic containers (OP1, OP2, OP3, OP4, IP1, IP3, and IP4) were equivalent in terms of harbouring both the species. IP1 and OP1 among the plastic types and OPR3 and OPR2 among the porcelain (glass) containerswere documented to sustain more *Aedes* spp. Probably the dimension of the varied household generated waste containers and the water retention ability/ residence time played a pivotal function in maintaining the abundance of the dengue vectors. The relative occurrence of the *Ae*. *aegypti* in the plastic containers was noted to be more than that of *Ae*. *albopictus* whereas the porcelain container types showed equivalence in holding both the species irrespective of the spots and the study period. Monthly variations in positive habitats and pupal productivity for both *Ae*. *aegypti* and *Ae*. *albopictus* were prominent both in the urban and rural spots of the study areas (Fig 1). Possibly due indiscriminate use of the plastic and porcelain materials in the urban areas as compared to the rural spots, the relative intensity of immature abundance of both the species was noted to be more in urban areas. Variation in the immature productivity may probably be an indication in the difference in generation of house-hold waste and thereby the extent of urbanization. In rural areas porcelain containers were preferred by *Ae*. *albopictus* compared to *Ae*. *aegypti*; while for plastic containers in both rural and urban spots *Ae*. *aegypti* seemed to be dominant contrasts to *Ae*. *albopictus*. Irrespective of locations, plastic containers were more productive with *Aedes* immature than glass containers, though significant variations among the months was evident (Fig 2A). Considerable variation in the number of pupa to larva was also noted during the period (Fig 2B). The post monsoon months, from July to October, were most productive perhaps due to accumulation and retention of water within the varied container types. Following a peak in August and September, the immature density declined gradually to almost nil in December. Five-way factorial ANOVA on the abundance of *Aedes* spp. revealed significant values for species, months, habitats, area and location of the habitats. Except for the interactions between species-location, habitat-area and month-habitat-area-location all other interactions were significant (Table 3A). The results of the 3-way factorial ANOVA on the positive habitats for *Ae*. *aegypt*i exhibited significant values for material, area and month and material-area interaction; for *Ae*. *albopictus* values for material and month were significant along with material-area, material-month, area-month and material-area-month interactions. Similar results were obtained from the 3-way factorial ANOVA on the abundance of the *Aedes* immature where material, area and month exhibited significant values for both the species. Significant interactions between material-area, material-month, area-month and material-area-month were noted for *Ae*. *aegypti* and material-area, material-month for *Ae*. *albopictus* (Table 4).

**Table 3:** Results of five-way factorial ANOVA (A) and Tukey test (B) on the abundance of *Aedes* immature considering species, months, habitats, area (urban-rural) and location of the habitats as variables. The F values significant at P < 0.05 level are marked in bold.

**A**						
Source			Sum of Squares	df	Mean Square	F
Species (S)			10862.89	1	10862.89	**657.44**
Month (M)			99432.40	5	19886.48	**1203.56**
Habitat (H)			15315.07	1	15315.07	**926.90**
Area (A)			369.44	1	369.44	**22.36**
Location (L)			487.53	1	487.53	**29.51**
S * M			5144.21	5	1028.84	**62.27**
S * H			9370.77	1	9370.77	**567.14**
S * A			228.01	1	228.01	**13.80**
S * L			16.73	1	16.73	1.01
M * H			6337.05	5	1267.41	**76.71**
M* A			606.02	5	121.20	**7.34**
M * L			417.83	5	83.57	**5.06**
H * A			49.62	1	49.62	**3.00**
H * L			89.59	1	89.59	**5.42**
A * L			186.58	1	186.58	**11.29**
S * M * H			2920.77	5	584.15	**35.35**
S * M * A			262.06	5	52.41	**3.17**
S * M * L			383.30	5	76.66	**4.64**
S * H * A			2098.55	1	2098.55	**127.01**
S * H * L			404.19	1	404.19	**24.46**
S * A * L			76.11	1	76.11	**4.61**
M * H * A			233.45	5	46.69	**2.83**
M * H * L			242.66	5	48.53	**2.94**
M * A * L			449.99	5	90.00	**5.45**
H * A * L			105.56	1	105.56	**6.39**
S * M * H * A			647.03	5	129.41	**7.83**
S * M * H * L			357.48	5	71.50	**4.33**
S * M * A * L			1302.35	5	260.47	**15.76**
S * H * A * L			150.33	1	150.33	**9.10**
M * H * A * L			112.77	5	22.55	1.36
S* M * H * A* L			302.82	5	60.56	**3.67**
Error			505983.26	30623	16.52	
Total			694727.94	30718		
**B** *Post hoc* Tukey test. Studentized range q = [|(I-J)|/S.E.] S.E. = 0.08; df = 3071, 5						
(I) Month	(J) Month	q		(I) Month	(J) Month	q
July	August	**2.64**		August	December	**4.31**
July	September	**0.96**		September	October	**1.28**
July	October	**0.32**		September	November	**2.64**
July	November	**0.32**		September	December	**4.69**
July	December	**1.67**		October	November	**1.36**
August	September	**3.73**		October	December	**3.41**
August	October	**1.67**		November	December	**2.06**
August	November	**2.95**				

**Table 4:** Results of three way factorial ANOVA on the positive containers of *Aedes aegypti* and *Ae*. *albopictus* immature and mean abundance considering material, spot (urban-rural spots), and months of the surveyed habitats as variables. The F values significant at P < 0.05 level are marked in bold.

***Ae*. *aegypti***					
		*Positive habitats*		*Immature abundance*	
**Source**	**df**	**Mean Square**	**F**	**Mean Square**	**F**
MATERIAL (M)	1	5260.55	**127.3**	2033839.17	**857.22**
SITE (S)	7	257.166	**6.221**	10960.91	**4.62**
MONTH (MTH)	5	12974.4	**313.9**	1169999.54	**493.13**
M * S	7	189.952	**4.595**	37549.14	**15.83**
M * MTH	5	146.097	3.534	138230.76	**58.26**
S * MTH	35	30.9052	0.748	6621.34	**2.79**
ML * S* MTH	35	29.6731	0.718	6026.44	**2.54**
Error	96	41.3385		2372.59	
Total	191				
***Ae*. *albopictus***					
		*Positive habitats*		*Immature abundance*	
**Source**	**df**	**Mean Square**	**F**	**Mean Square**	**F**
MATERIAL (M)	1	744.19	**29.45**	59925.33	**13.50**
SITE (S)	7	24.87	0.98	9700.58	**2.19**
MONTH (MTH)	5	15194.73	**601.28**	897908.87	**202.33**
M * S	7	80.40	**3.18**	17602.98	**3.97**
M * MTH	5	289.66	**11.46**	17621.62	**3.97**
S * MTH	35	72.09	**2.85**	2354.36	0.53
ML * S * MTH	35	73.99	**2.93**	4685.08	1.06
Error	96	25.27		4437.84	
Total	191				

**Fig 3:**
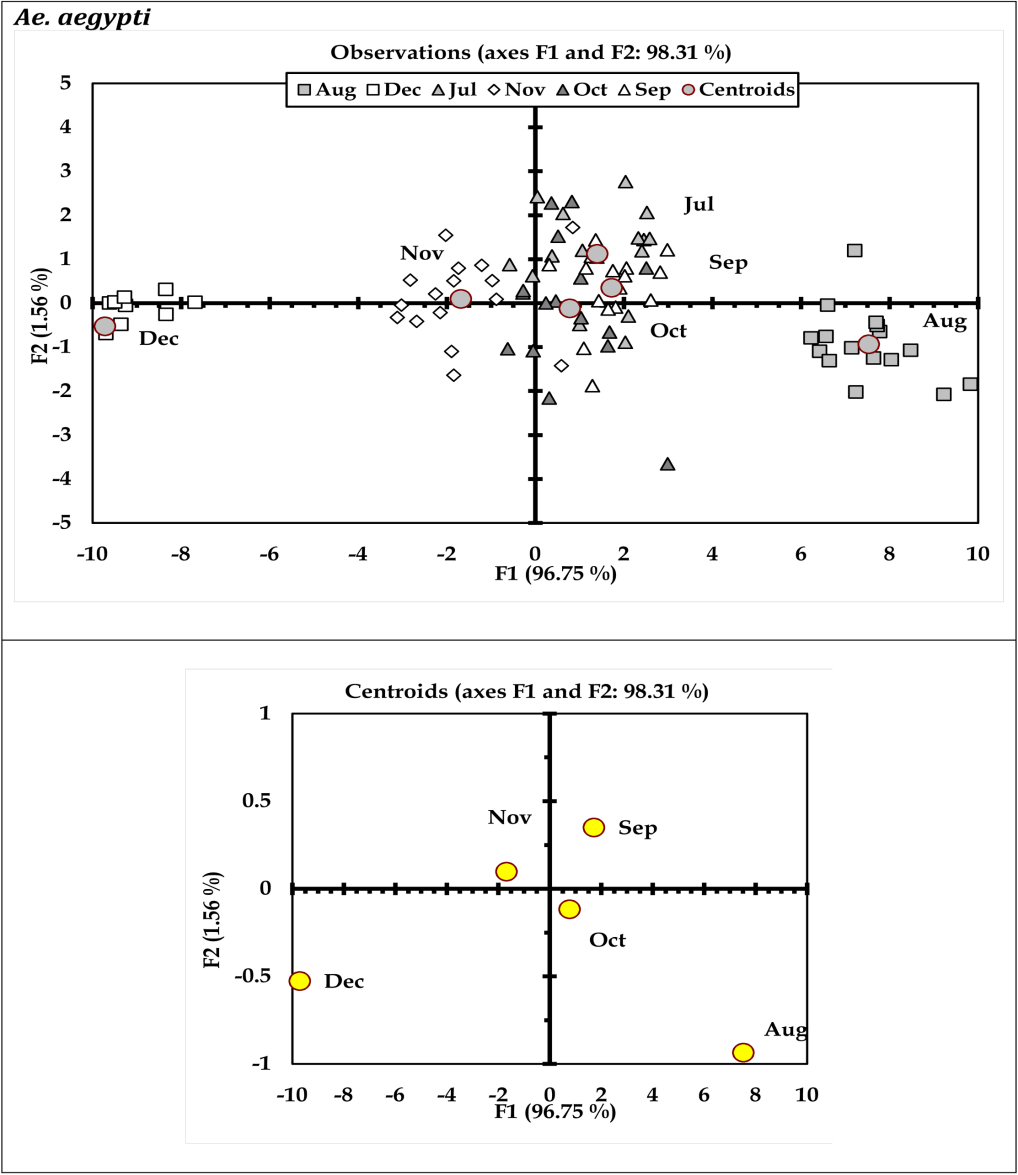
Biplot representing the ordination of sampling months in terms of *Ae*. *aegypti* productivity in the surveyed household wastes (Wilk’ λ = 0.015; F_30, 342_ = 21.15; P<0.0001).

**Fig 4:**
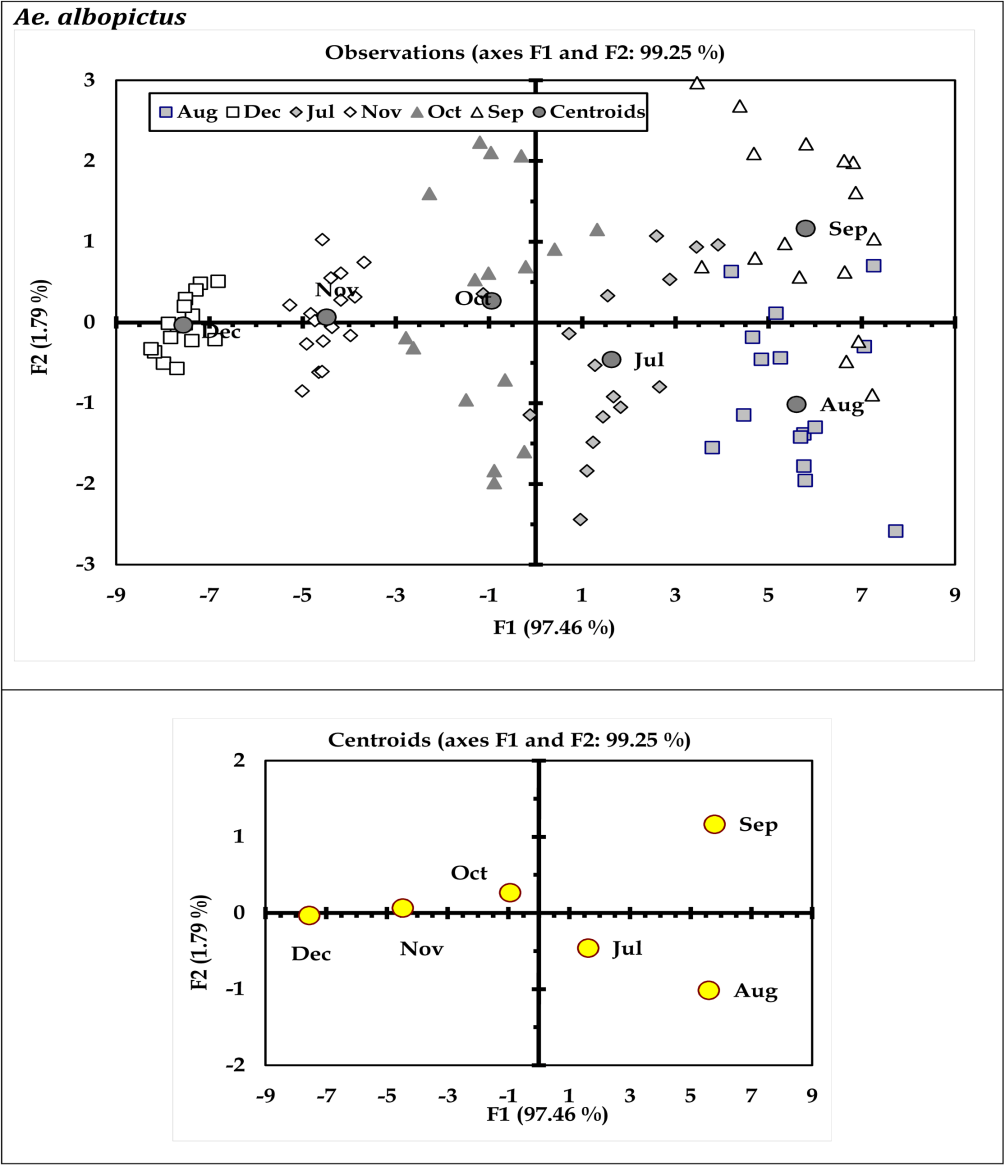
Biplot representation of the ordination of sampling months in terms of *Ae*. *albopictus* productivity in the surveyed household wastes (Wilk’ λ = 0.021; F_30, 342_ = 180576; P<0.0001).

**Fig 5:**
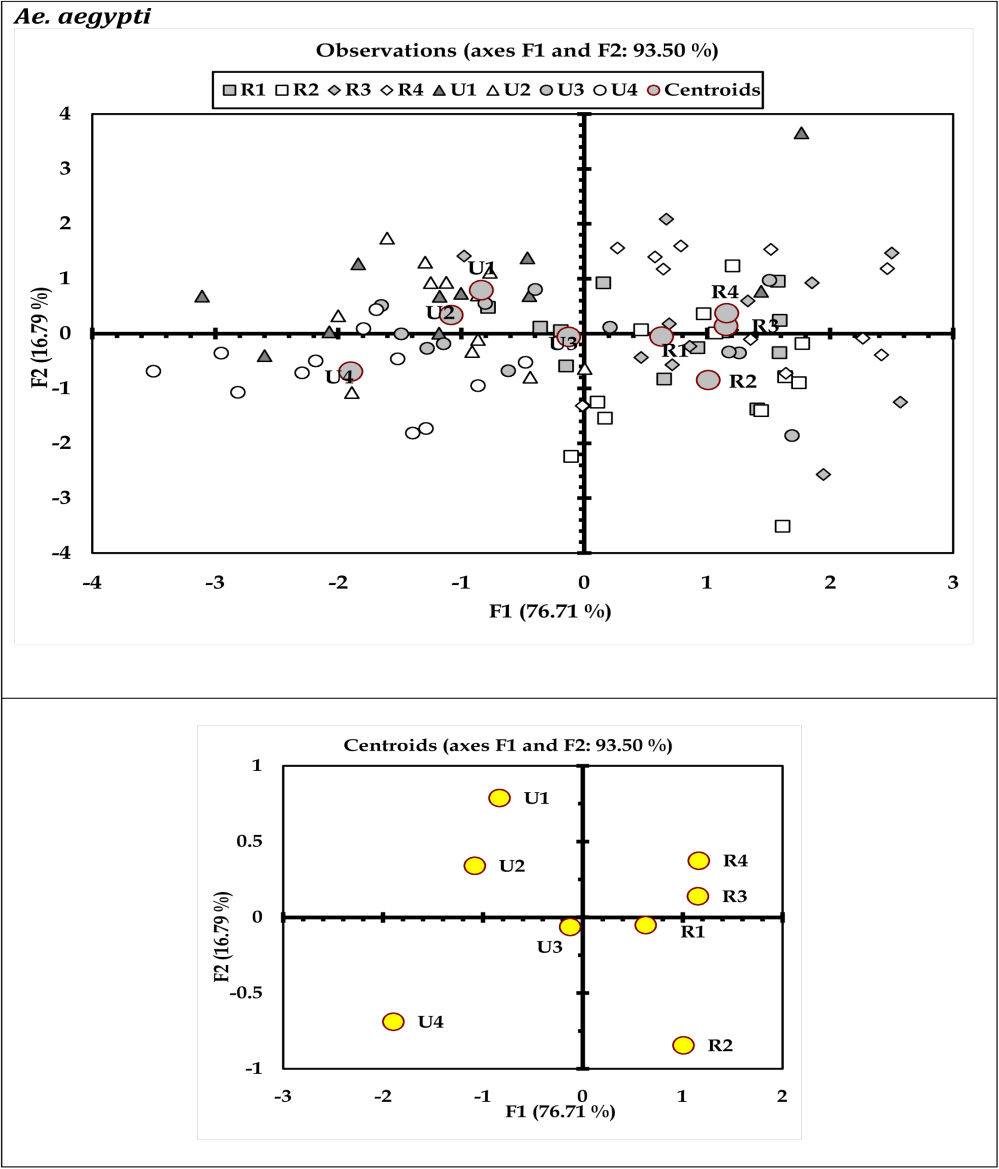
Biplot representation of the ordination of urban-rural areas in terms of productivity of *Ae*. *aegypti* in the household wastes surveyed(Wilk’ λ = 0.0303; F_42, 393_ = 2.708; P<0.0001). Here, R (rural)1 = Serampore, R2 = Baidyabati, R3 = Singur and R4 = Haripal and U (urban)1 = Baranagar, U2 = Ballygunge, U3 = Chetla, and U4 = Patuli.

**Fig 6:**
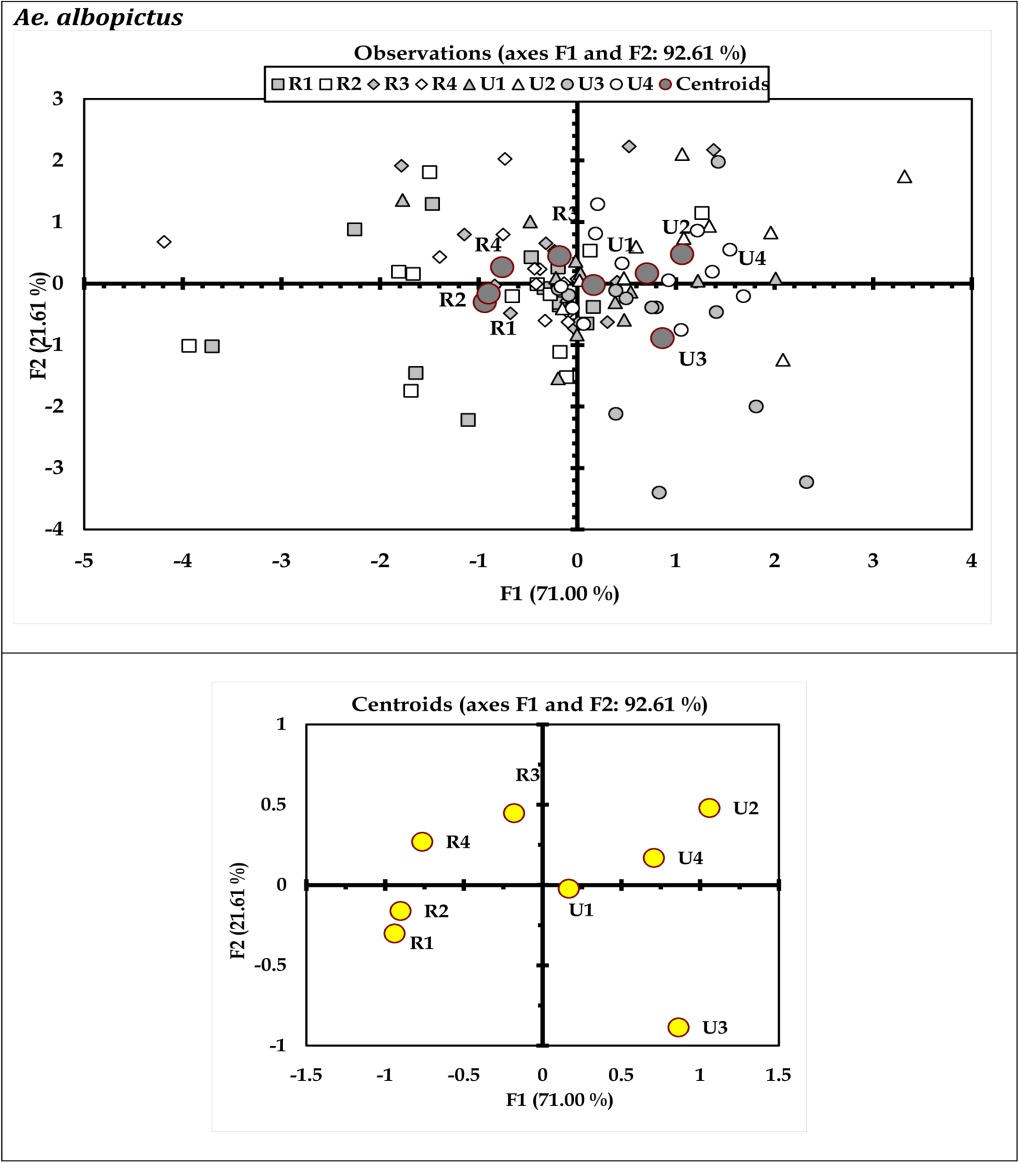
Biplot representation of the ordination of urban-rural areas in terms of productivity of *Ae*. *albopictus* in the household wastes surveyed (Wilk’ λ = 0.477; F_42, 393_ = 1.589; P<0.013). Here, R (rural)1 = Serampore, R2 = Baidyabati, R3 = Singur and R4 = Haripal and U (urban)1 = Baranagar, U2 = Ballygunge, U3 = Chetla, and U4 = Patuli.

Results of discriminant analysis (DA) indicated variations in immature productivity of *Ae*. *aegypti* and *Ae*. *albopictus* with respect to months (Figs 3 and 4) and urban-rural scenario (Figs 5 and 6). These characteristic differences between the urban and rural sites reflect prospective differences in the glass and plastic wastes generation and subsequent conversion as larval habitats. The discriminant function coefficients were derived from the construction of sum of squares and cross product matrices of the explanatory variables. The coefficients represent the contribution of the variables against the three discriminant functions (F1, F2 and F3; Tables 5 through 8). The canonical correlation coefficients for each of the discriminant functions (F1 through F3) represent the strength of the overall relationship between a variate for the independent variables (immature productivity) and one for the dependent variables (Sites or months as applicable). For both the mosquito species *Ae*. *aegypti* and *Ae*. *albopictus*, the Fisher’s distances were found to be significant (P < 0.05) with respect to months (Tables 5 and 6) and urban-rural areas as well (Tables 7 and 8). The ordination of the variables (months and sites) along the biplot axes represent sufficient discrimination of the months and urban-rural sites based on their abundance for both the species.

**Table 5:** The results of Discriminant Analysis showing Fishers distance, standardized canonical correlations and Eigen values of the months and explanatory variables, in case of *Ae*. *aegypti*. Porcelain and plastic habitats are denoted by pr and pl.

*Ae*. *aegypti* -Month					
Fishers distance	Aug	Dec	Jul	Nov	Oct
Dec	**374.828**				
Jul	**52.748**	**159.120**			
Nov	**109.582**	**83.661**	**15.215**		
Oct	**60.392**	**140.283**	**4.854**	**10.752**	
Sep	**45.902**	**166.566**	**2.554**	**17.050**	**1.611**
Standardized canonical discriminant function coefficients					
			F1	F2	F3
Positive pr			0.257	0.630	0.366
Larvae pr			0.598	-0.175	-0.907
Pupae pr			-0.043	-0.382	0.580
Positive pl			0.519	0.445	-0.006
Larvae pl			0.382	-0.505	-0.115
Pupae pl			0.286	-0.271	0.732
			F1	F2	F3
Eigen value			28.345	0.456	0.305
Discrimination (%)			96.752	1.557	1.042
Cumulative %			96.752	98.309	99.35
Canonical correlations			0.983	0.560	0.484

**Table 6:** The results of Discriminant Analysis showing Fishers distance, standardized canonical correlations and Eigen values of the months and explanatory variables in *Ae*. *albopictus*. Porcelain and plastic habitats are denoted by pr and pl.

*Ae*. *albopictus*-Month					
Fisher distances	Aug	Dec	Jul	Nov	Oct
Dec	**219.374**				
Jul	**22.104**	**107.852**			
Nov	**129.626**	**12.158**	**48.602**		
Oct	**56.649**	**55.841**	**9.407**	**16.102**	
Sep	**6.155**	**226.379**	**26.217**	**134.638**	**58.483**
Standardized canonical discriminant function coefficients					
			F1	F2	F3
Positive pr			0.579	-0.287	0.605
Larvae pr			0.184	-0.510	0.118
Pupae pr			0.097	0.286	-0.232
Positive pl			0.586	0.777	0.212
Larvae pl			0.230	-0.749	-0.294
Pupae pl			0.184	0.198	-0.614
			F1	F2	F3
Eigen value			25.903	0.475	0.173
Discrimination (%)			97.460	1.788	0.650
Cumulative %			97.460	99.248	99.898
Canonical correlations			0.981	0.568	0.384

**Table 7:** The results of Discriminant Analysis showing Fisher’s distance, standardized canonical correlations and Eigen values of the urban-rural areas and explanatory variables in *Ae*. *aegypti*. Porcelain and plastic habitats are denoted by pr and pl.

***Ae*. *aegypti***					
		*Positive habitats*		*Immature abundance*	
**Source**	**df**	**Mean Square**	**F**	**Mean Square**	**F**
MATERIAL (M)	1	5260.55	**127.3**	2033839.17	**857.22**
SITE (S)	7	257.166	**6.221**	10960.91	**4.62**
MONTH (MTH)	5	12974.4	**313.9**	1169999.54	**493.13**
M * S	7	189.952	**4.595**	37549.14	**15.83**
M * MTH	5	146.097	3.534	138230.76	**58.26**
S * MTH	35	30.9052	0.748	6621.34	**2.79**
ML * S* MTH	35	29.6731	0.718	6026.44	**2.54**
Error	96	41.3385		2372.59	
Total	191				
***Ae*. *albopictus***					
		*Positive habitats*		*Immature abundance*	
**Source**	**df**	**Mean Square**	**F**	**Mean Square**	**F**
MATERIAL (M)	1	744.19	**29.45**	59925.33	**13.50**
SITE (S)	7	24.87	0.98	9700.58	**2.19**
MONTH (MTH)	5	15194.73	**601.28**	897908.87	**202.33**
M * S	7	80.40	**3.18**	17602.98	**3.97**
M * MTH	5	289.66	**11.46**	17621.62	**3.97**
S * MTH	35	72.09	**2.85**	2354.36	0.53
ML * S * MTH	35	73.99	**2.93**	4685.08	1.06
Error	96	25.27		4437.84	
Total	191				

**Table 8:** The results of Discriminant Analysis showing Fishers distance, standardized canonical correlations and Eigen values of the urban-rural areas and explanatory variables in *Ae*. *albopictus*. Porcelain and plastic habitats are denoted by pr and pl.

*Ae*. *aegypti*- Area							
Fisher’s distance	R1	R2	R3	R4	U1	U2	U3
R2	**0.263**						
R3	**1.080**	**1.146**					
R4	**0.697**	**0.255**	**0.786**				
U1	**1.300**	**1.166**	**0.438**	**1.009**			
U2	**4.414**	**4.101**	**1.571**	**3.344**	**1.006**		
U3	**3.469**	**3.497**	**2.822**	**3.863**	**1.164**	**1.818**	
U4	**3.049**	**2.591**	**1.137**	**2.120**	**0.420**	**0.376**	**1.158**
Standardized canonical discriminant function coefficients							
		F1		F2		F3	
Positive pr		1.911		1.945		1.138	
Larvae pr		-2.205		0.410		-0.583	
Pupae pr		-0.556		-0.577		0.260	
Positive pl		0.556		-0.573		0.314	
Larvae pl		0.110		-2.388		2.312	
Pupae pl		0.059		1.113		-3.536	
		F1		F2		F3	
Eigen value		0.642		0.196		0.055	
Discrimination (%)		70.999		21.611		6.040	
Cumulative %		70.999		92.610		98.650	
Canonical correlation		0.625		0.404		0.228	

All the data set for the tables and figures have been provided in the supporting information file (S1 File).

## Discussion

Surveillance of *Aedes* mosquitoes in rural and urban areas around Kolkata, India, reveals that the household plastic and glass wastes contribute to the existence of the dengue vectors to a considerable extent. The number of plastic and glass wastes serving as larval habitats of *Ae*. *aegypti* and *Ae*. *albopictus* varied among the months in both rural and urban areas. Exploitation of glass and plastic wastes as breeding sites varied between indoor and outdoor locations of urban and rural areas, perhaps due to differences in the anthropogenic activities and thus generation of household wastes. The observations of the present study is a pioneer effort to highlight the importance of rural areas as potential dengue breeding sites in the context of Kolkata and adjoining areas of India. Although rural areas were featured by fewer number of wastes as *Aedes* larval habitats than urban areas, the consistency in the immature density through the months, calls for consideration of rural areas as prospective sites for breeding of dengue vectors and thus possibilities of dengue. Until now, resurgence of dengue and corresponding vector management strategies are focused on urban areas of India [25–27,43,44], though few studies have demonstrated the pattern of dengue vector abundance in rural areas [29,45,46]. In rural areas, the alternative breeding habitats of mosquitoes like tree holes and puddles are quite common [35,47,48]. Increased availability of the household wastes in rural areas will increase the potential breeding sites of *Aedes* mosquitoes, as well as risk of dengue in rural areas in and around Kolkata, India.

Improper usage and inappropriate disposal of various commodities of daily use including different articles and containers made up of plastic pose a menace to the public health [49]. Due to high durability, low cost, and versatile forms, plastics and allied products have become an indispensable part of modern life. Resistance to microbial and physical degradation routes however, enables plastic wastes to act as environmental nuisance [50]. Porcelain (Glass) containers featured by frost-resistant and radiant glazes are non-biodegradable. Owing to slow degradation by physical means, the residence time of glass wastes in environment increases the burden of waste in environment. Improper disposal and management increases the possibilities of plastic and glass wastes to serve as breeding habitats for containers breeding mosquitoes, *Aedes* in particular. As observed in the present study, the pupal productivity varied with the shape and size of the plastic and glass waste containers, similar to the observations made in Australia [51], Africa [52], Vietnam [53], North and South America [54–58]. It seems that identification and classification of plastic and glass household wastes needs to be reviewed in terms of hazard potential and adverse health impact in both urban and rural areas [21,59,60].

The present survey was carried out in the urban and rural regions of Kolkata metropolis as model geographical region with the aim to identify and classify those hazardous containers responsible for sustaining *Aedes*. Seasonal variations and periodicity was amply reflected in the relative abundance of the *Aedes* immature in the waste containers similar to many other places around the globe [9,12,17,23]. Preference of the dengue vectors for the waste container, based on the location and type of material, could be deduced through the corresponding immature productivity (Figs 1 and 2). The monthly variations in the relative abundance of *Ae*. *aegypti and Ae*. *albopictus* as reflected in the biplots (Figs 3 and 4) can be attributable to the differences in the availability of congenial breeding sites. Although *Aedes* can exploit varied kind of plastic and glass wastes as larval habitats, the availability of such wastes in itself is a major concern for vector management. Population regulation of *Ae*. *aegypti* and *Ae*. *albopictus* is constrained primarily due to its exploitation of domestic environment for breeding and secondarily due to human-mediated dispersal that enhances abundance at spatial scale [48,52,54]. Mosquito productivity increases with the availability of the porcelain (including glass) and plastic waste containers, and thus appropriate measures should be taken to reduce the waste generation and management [61–63]. Inappropriate use of the porcelain and plastic containers along with poor waste management strategies lead to an extended life of the porcelain and plastic waste products. Variation in waste generation in space leads to the diversification of the breeding sites of *Aedes* mosquitoes, thereby leading to surge in mosquito abundance. Possibly the generation of the wastes varied over the months contributing to the differences in the abundance of the mosquitoes in the area. Persistence of such waste products adds to the permanence of breeding and growth of *Aedes* mosquitoes and thus the possibility of dengue episodes [64]. Effective solid waste management strategies for Kolkata [19,65] and other similar cities where household wastes are contributing to mosquito breeding [66,67] should be prioritized for intervention of *Aedes* population and reduce the risk of dengue and chikungunya.

Water retention capability and resource content enable porcelain (including glass) and plastic containers as favourable breeding habitats of dengue vectors. In urban areas, the frequent disposal of household plastic wastes is common contrast to the rural areas [31,32]. In Indian context, waste generation is linked with the socioeconomic factors [30], which are expected to differ between urban and rural communities. Although socioeconomic and life style patterns differ between urban and rural areas, it appears that the generation of waste in rural areas differ from urban areas quantitatively but not qualitatively. As a result, the breeding grounds of dengue vectors were more abundant in urban areas, with higher frequency of dengue incidence, as portrayed in the biplots (Figs 5 and 6). However, the present study suggests that the trend may change, since the porcelain and plastic wastes generated in rural areas are equally compatible for *Aedes* breeding. Thus vector control strategies should incorporate the rural and suburban areas for regulation *Aedes* mosquito abundance. While only few discrete studies in rural areas of North India [19,43,45] record the occurrence of the dengue vectors, planned dengue vector control strategies are yet to be employed. In majority instances in India and other tropical regions, dengue vector control is restricted to urban populated sites [4,45, 49]. Extending the previous observations on dengue vectors in rural areas through the present study, refined strategies may be framed for dengue vector control inclusive of rural areas. Accessibility of waste containers in indoor or outdoor locations depends on knowledge and attitude of the people about likelihood of waste into prospective *Aedes* larval habitat [11,15]. Appropriate management practice can reduce availability of waste containers, thereby reducing the prospective *Aedes* larval habitats. Possible limitations in such practices may have allowed conversion of porcelain and plastic wastes as larval habitats in the present study area. The regulation or local elimination of dengue vectors are often limited by the recurrent colonization in respective habitats following availability of resources and water. The ability of the eggs of *Aedes* mosquitoes to withstand desiccation is another factor that can facilitate re-colonization in the same habitat following control. While natural larval habitats like tree hole can rarely be modified, restriction of the waste generated forms a major way of creation of habitats for larval breeding. Appropriate steps may therefore be taken to reduce the generation of the plastic and porcelain (including glass) wastes along with scientific methods for disposal so that the reduction of the sources of breeding is ensured. The citizens should be communicated about the potential harm owing to these wastes, as well [33,68]. It is pertinent to mention that the present study is limited in terms of exploring all the possible habitats of dengue vectors, including the tree holes and bromeliads. The strategies for vector management should include such habitats where the pupal productivity of *Aedes* mosquitoes is equally a concern for public health. Restriction of breeding of *Aedes* mosquitoes is of prime importance to reduce the incidence of dengue and chikungunya. In order prioritize larval habitat based population intervention to reduce possibilities of dengue, studies may be initiated to determine the pattern and preference of oviposition habitats by *Aedes* mosquitoes in urban and rural areas of India and Kolkata in particular.

The entire data set, tables (Tables 1 through 8), and figures (Figs 1 through 6) used in the present manuscript are included in the supporting information files.

## Supporting Information

S1 File This file contains the entire data set related to Tables 1through 8 of the present manuscript. (XLSM)

S2 File This file contains the entire data set related to Fig 1 and Fig 2 of the present manuscript. (XLSM)

S3 File This file contains the entire data set related to Figs 3 through 6 of the present manuscript. (XLSM)
